# ‘One Stroak of His Razour’: Tales of Self-Gelding in Early Modern England

**DOI:** 10.1093/shm/hky100

**Published:** 2018-11-14

**Authors:** Alanna Skuse

**Keywords:** gender, disability, sexuality, mental health, surgery, self-harm

## Abstract

This article examines stories of men who gelded themselves in early modern England. These events, it argues, were shaped and partly motivated by a culture in which castration was seen as both degrading and potentially empowering. Religious precedents such as that of Origen of Alexandria framed self-gelding as a foolhardy activity, but one which nevertheless indicated an impressive degree of mastery over the body and its urges. Meanwhile, judicial and popular contexts framed castration as a humiliating and emasculating ordeal. Instances of self-gelding in this period are rare but nonetheless illuminating. Relayed in medical texts and popular ballads, such actions typically occurred as a response to emotional distress. In particular, men gelded themselves as a means to express feelings of emasculation within heterosexual relationships, and to dramatically renounce their role in the libidinal economy.

In 1676, the surgeon James Yonge was called to an unusual kind of accident. A young man of about twenty, the nephew of ‘alderman W.’, was bleeding dangerously from the groin. This in itself was fairly uncommon, and potentially fatal. When Yonge arrived at the scene, he was surprised to discover that the injury was self-inflicted:

from some disappointment in Love, as was imagined, or rather as himself confessed, on a Religious account, to cure salacious heats, [the patient] did castrate himself, by griping up the *Testicles*, with the whole *Scrotum* in one hand, and with a keen Knife in the other cutting them off close to the body; the sudden pain and effusion of bloud made him faint and fall back on the Bed, where he sat while he thus acted *Origen Secundus*.^1^

Yonge’s patient had ‘bled very largely before any one discovered it’, and was in a perilous state. In his *Currus Triumphalis*, Yonge described how he gripped the wound in his hand in order to stop the haemorrhage while his assistant prepared dressings; when he withdrew his hand, he recalled, ‘the bloud forthwith spouted out, as it had been from a small quill’.^2^ Between Yonge and his colleague, the bleeding was got under control, but the patient, suffering from blood loss and shock, fainted. Observing his coldness and lack of discernible pulse, Yonge concluded that death was imminent. Nonetheless, they managed to give the patient a glass of sack and a medicine composed of hyacinth, crocus, alkernes, mirabilis and Melissa. Soon he began to recover, and when the surgeons returned the following day they found:

tokens of good Digestion, and as fair as heart could wish. It was long e’re he could recruit his spirits, so much exhausted by the *Hemoorrhage*; but the Wound in a month’s time was almost cicatrized, so as he followed his business.^3^

Yonge was a skilled medical practitioner, and his quick action to staunch blood loss probably saved the patient’s life. Why, though, would the alderman’s nephew have chosen to mutilate himself in this way? Was his claim of religious fervour genuine, or was it, as Yonge suspected, a smokescreen for ‘some disappointment in Love’? If so, what did he hope to achieve by this extreme variety of self-harm?

This article will examine several accounts of self-gelding in early modern England, from both medical and popular literary texts. It argues that self-gelding offered individuals in emotional distress a means of expressing their feelings and attempting to gain relief. Moreover, this particular mode of self-harm had a cultural significance shaped by the history of religious and judicial castration and gelding. Castration in these contexts could be humiliating, terrifying and sometimes empowering. Early modern tales of self-gelding often framed the event as grimly humorous, but they also hinted at a complex psychodrama. To geld oneself was a curiously masochistic response to fears of cuckoldry or conjugal disorder. At the same time, it was a repudiation of the body and its urges; both an attempt at self-determination and an annihilation of the masculine subject.

It should be noted that in this article I use the term ‘gelding’ to describe the removal of the testicles, unless otherwise specified. Amputation of the penis was occasionally carried out in this period for medical reasons, as well as in a few instances as a form of judicial mutilation. However, those who suffered such amputations usually died, and instances of penile amputation on any account were exceedingly rare.^4^

## Acting ‘*Origen Secundus*’: Gelding in the Cultural Imaginary

Could the alderman’s nephew really have acted on a ‘religious account’ when he decided to geld himself? Yonge himself was dubious about this purported motive, but his patient’s claim was not entirely baseless. The Bible mentions eunuchism in several places, and its most famous statement on the matter seemed positive about the spiritual advantages of castration:

For there are some eunuchs, which were so born from their mother’s womb: and there are some eunuchs, which were made eunuchs of men: and there be eunuchs, which have made themselves eunuchs for the kingdom of heaven’s sake. He that is able to receive it, let him receive it. (Matthew 19: 12, KJV)

On the strength of these few lines, several famous religious figures either framed their desire for castration or—in the most extreme cases—decided to geld themselves. Probably the earliest instance of this idea appeared in Justin Martyr’s *Apology* (*c*.155–157), in which the author wrote with sympathy about a young believer who had (unsuccessfully) sought permission to be gelded in order to dispel rumours about sexual licentiousness among Christians.^5^ However, the most prominent self-gelder (and the example which sprang to Yonge’s mind) was the religious scholar Origen of Alexandria (*c*.184–253). Eusebius’s *Church History* (*c*.323) reports that Origen, spurred on by the above verse, gelded himself because ‘he was eager to fulfil the Savior’s words and also to forestall all slander on the part of unbelievers’.^6^ As he was involved with the teaching of women, it was alleged, Origen sought to quash all suspicions of a sexual motivation for his activities. Origen initially tried to conceal his orchiectomy; however, Eusebius states, ‘Demetrius [Bishop of Alexandria] learned of it later, since he presided over the community there. He was astonished at Origen’s rash act but approved the genuine enthusiasm of his faith.’^7^

Whether reports of Origen’s gelding had any basis in truth remains unclear.^8^ Nonetheless, as Mathew Kuefler points out, ‘men who lived after Origen believed that he had castrated himself’.^9^ Eusebius’s version of events was apparently accepted as fact in the medieval and early modern period, during which several translations of the *Church History* were printed.^10^ References to Origen’s story appear in medical and popular texts as well as religious works, indicating the place of this tale in the popular consciousness. The majority of early modern writings on Origen dismissed his self-castration as a well-intentioned but foolish act, based on a too-literal interpretation of scripture. The 1622 *The Christian’s Sacrifice* was typical:

*Origen* (though in other things too much allegoricall) interpreting the words of our Saviour literally, *There be Eunuches which have made themselves Eunuches for the kingdome of heaven; Origen* (I say) upon the said words literally interpreted, did geld himselfe. … But it was not thus from the beginning. For God at the first creation of man, said, *It is not good for man to be alone.*^11^

Likewise, *The new command renew’d* (1652) advised readers that they ought to ‘*Distinguish between things spoken properly, and things spoken figuratively*’.^12^ Origen, noted the author, had ‘interpreted … *almost all* other places of Scripture mystically’, yet gelded himself based on an over-literal reading of Matthew 19: 12.^13^

In their antipathy to gelding, writers from the English Protestant church followed the example which had long been set by Catholic leaders.^14^ The Council of Nicaea (Ad 325) and the *Apostolic Constitutions* (*c.*ad 375) banned self-gelded men from becoming clergy, and decreed that self-gelding laity be punished with three years excommunication. Writing on castration in early Christianity, Daniel F. Caner reiterates that self-gelding was viewed as at best, misguided, and at worst, instigated by the devil, who exploited people’s desire to avoid sin.^15^ For instance, in the twelfth-century *Codex Calixtinus*, the devil, disguised as Saint James, appears to an unchaste pilgrim and persuades him to castrate himself (which on this occasion included severing his penis). Ryan Giles summarises the tale:

The pilgrim, Gerald, fornicates with a ‘maiden’ before starting his journey. On the road, the devil sends a demon to remind Gerald of his unconfessed state and recommend that he emasculates and kills himself. Gerald severs his genitals and gores himself. When he is dragged to church (dead) for burial, he comes back to life and says how the real St. James appealed to the Virgin Mary to save his soul and body. He goes on to complete the pilgrimage and tell his story. According to the narrator, ‘he retold the story, showed his scars and even showed what had been in his most private place to the many people wanting to see it’.^16^

The message in all these accounts was clear. Physical castration represented both the mutilation of God’s creation, and an attempt to short-cut one’s way to moral fortitude by removing the capacity to sin. In spite of this, even the fiercest critics of self-castration had to admit that the desire to avoid sin was basically a good thing. While Eusebius described Origen as immature, he conceded that his actions demonstrated considerable ‘self-mastery’.^17^ Accordingly, both Giles and Caner recognize that castration held a certain glamour for those eager to be rid of troublesome desires.^18^ The tale of Hugh of Lincoln epitomised this appeal. To avoid sexual temptation, Hugh cut out a portion of his arm after it was touched by an attractive woman. Later, it was said, he prayed for assistance in resisting temptation once more. According to different versions of the story, either a deceased prior of the monastery he inhabited, or an angel, came to him in a vision and gelded him. This mystical operation freed Hugh once and for all from his sinful desires. Saint Hugh’s story had an altogether more positive slant than that of Origen. Indeed, *mystical* castrations of this sort seemed like the ideal solution to the problem of sexual temptation.^19^ Thus, as Giles notes, ‘There can be little doubt that such legendary visions of stylized, redemptive violence, accompanied by warnings “not to try this at home” would have influenced the development of a negative exemplar of self-castration as part of the Jacobian miracle tradition.’^20^ Furthermore, even when castration was mystical, it still ‘locat[ed] the site of a man’s spiritual struggle and his identity in his genitals’.^21^ Regardless of the proscriptions against it, self-castration still possessed connotations of holy fervour. It may have been these connotations which allowed some within the Catholic Church to embrace the use of castrato singers in Church music, though boys gelded for this purpose still usually claimed to have met with some accident which caused their emasculation.^22^ Interestingly, however, this kind of gelding seems to have had little impact on how ‘ordinary’ men perceived losing their testicles. Gelding children created adults with a range of unusual characteristics, and this, combined with the foreignness of castrato singers, made them something more like a different species in the public imagination.

Despite the agency that accompanied some varieties of religious castration, it remains the case that most non-medical castrations were understood as punitive. The history of judicial castration is well-documented; work on this subject has been undertaken by Martin Irvine and Lila Yawn, among others.^23^ Here, it is worth noting how judicial castration, either as part of an execution or as a punishment in its own right, diametrically opposed the reclamation of bodily agency which seemed—however abstractly—to accompany some instances of religious self-castration. Removal of the scrotum, penis or both as punishment was explicitly designed to humiliate and torture the victim, and as such, was generally only included in the executions of particularly notorious criminals and traitors, alongside such practices as disembowelling, quartering and burning. For instance, Murray notes that in the medieval period, castration was one of the most common punishments meted out to men found guilty of committing homosexual acts.^24^ During such punishments the testicles of the offender might be burned or tossed into the crowd to further their humiliation.

Revenge for sexual misdemeanour also underpinned the famous mutilation of Peter Abelard (1079–1142), whose ill-fated romance with Heloise d’Argenteuil was well-known in the early modern period. An iconoclastic religious scholar, Abelard began an affair with Heloise while they were both living in Paris in the house of Heloise’s uncle, Fulbert. When Heloise became pregnant, Abelard sent her to his family in Brittany to give birth, and proposed marriage, which she reluctantly accepted. When upon being questioned by Fulbert, Heloise denied the marriage, Abelard sent her to a convent in Argenteuil to avoid her uncle’s rage. Unfortunately, Fulbert interpreted this move as an attempt by Abelard to get rid of Heloise. Abelard was accosted at night by Heloise’s uncle and his henchmen, and his testicles removed in a sequence of events that, as Irvine points out, mirrored those of a rape.^25^ The attack was explicitly designed to humiliate Abelard, and it initially worked.^26^ He entered a monastery and persuaded Heloise to become a nun. At length, however, he exploited his castrate status to depict himself as intellectually and spiritually superior.^27^ Notably, Abelard’s attackers, when apprehended, were subjected to both castration and blinding. This was arguably the closest possible thing to castrating the men twice over, since blinding and gelding were so closely associated.^28^ Moreover, such a punishment reinforced the parallel between Abelard’s ordeal and rape; as Karin Sellberg and Lena Wånggren point out, medieval penalties for rape might require the rapist ‘to lose his eyes as well as his testicles because the eye was held responsible for inspiring uncontrolled sexual desire’.^29^

While public displays of legal violence waned in the early modern period, the association between gelding, punishment and ignominy remained. In 1731, for example, it was suggested that thieves be punished by gelding rather than branding or hanging. The reasons advanced for this proposition were various: the punishment would be a deterrent; it would prevent the influence of ‘lewd women’; it would eradicate criminal bloodlines; and it would produce men potentially useful as singers or caretakers for noble wives and daughters.^30^ Foremost, however, was the notion that the gelded thief would be continually shunned and humiliated by his community and by strangers.

The pains of hanging are quickly over, and the death and name of the party are soon forgotten; whereas the circumstances of castration will remain as a living monument of shame and disgrace, and will more effectually deter people from those crimes for which they suffer’d, than if they had been hung in chains on a gibbet, where the body and bones continue but a short time, and that only in one place, while the gelded thief will sometimes be obliged to travel from town to town, where the mob will follow him as a May-game, or an outlandish monster.^31^

The continued humiliation of the castrated man relies in part on the relative difficulty of reinventing oneself in this period. The marks of gelding are not visible when one is clothed, but the author seems confident that this gelded criminal will remain in his community or travel only to places where members of the ‘mob’ may recognise him and tell others about his shame. As an ‘outlandish monster’, the gelded man in this narrative is also dehumanised—a pattern also visible when the author cited a grim precedent for this form of punishment in the mutilation of rebel slaves in Barbados. To avoid executing the slaves and losing their labour, he reported, ‘a great number of the rebellious slaves were ordered, like some wild vicious cattle, to suffer the punishment of castration; … This struck such terror in the minds of the blacks, that we never heard that they attempted any more rebellions.’^32^ In that case, as in this, gelding was viewed as a sort of capital punishment without death, a means to annihilate the victim as an independent subject without losing his economic value.

Gelding in judicial terms was thus firmly a means of humiliation, in which the impulse to disempower the victim was prominent. In this guise it also appeared in extrajudicial contexts, where removal of the penis, testicles or both was often threatened as revenge for sexual misdemeanours. Here, popular ballads picked up the theme of retributive castration where formal justice left off; they almost always represented gelding as an amusing variety of rough justice, in which the victim usually had it coming. The late seventeenth-century ballad ‘Robin the Plowman’s courage’ is typical of this narrative.^33^ As the ballad opens, Harry the Miller is attempting to woo Kate, who declares that she will never marry him. Robin the Plowman enters and starts arguing with his love rival; whereupon Kate reveals that she despises Harry because she knows him to be castrated:

remember the time that Bridget held,
While Margery, Nancy, Jone, and Kate,
 did for your wicked Actions geld you,
Therefore, therefore never stand contending,
 since I the truth have thus related,
Where e’er you go, ye Rascal ye know,
 you are by all young women hated.
The Miller at this did blush for shame,
 and happy he was to sneak away,
While Robin the Plow-man he by Name,
 with conquering courage gain’d the day.

Several elements here contrive to make Harry’s humiliation complete. His gelding is specified to have taken place at the hands of women. By withholding her knowledge until Robin is present and the two men are on the point of fighting, Kate undermines the miller’s attempt to assert his masculinity through violence. Finally, we learn that his castration becomes common knowledge throughout the local area, such that ‘Now for his life, he cannot get a wife’. Despite these misfortunes, the miller is firmly painted as the villain of the piece; his castration is symptomatic of his bad character.

There is a similar sense of retributive justice in two 1680s ballads on the theme of the ‘Hertfordshire Maidens’. In the first of the pair, ‘The Nine Maidens Fury to the Hartford-Shire Man’, a gang of woman capture and restrain a young man. They are resolved to geld him as punishment for his sexual misdemeanours:

The reason why they would do so,
 he alwaies would be fooling
Where ever he did come or go,
 His Courage wanted cooling:
He was for trimming both maids & women
 they ran if they beheld him
But now stout Doll, with Kate, and Moll,
 are all resolv’d to geld him.^34^

It is unclear from the above lines whether their victim’s crimes include sexual violence or merely lascivious conduct, but the young man is on the point of losing his testicles when the gang are met by his lover, Susan. After Susan pleads with the attackers, ‘Jone vow’d she’d save what Sue should have / therefore she would not geld him’. Notably, Susan’s presumed ownership of her lover’s body here inverts the usual rules of coverture, in which wives were the possessions of their husbands. Foiled on this occasion, the gang are apparently not put off their quest, for the subsequent ‘Hartford-shire Mens Fears of the Maidens Furies’ describes what is presumably the same gang of women roaming the Hertfordshire countryside looking for young men to geld:

I Wonder that this Age is grown
 to such a vast confusion;
That maids won’t let young-men alone,
 but by a strange Intrusion
They take much pleasure to gain their treasure
 their very fingers itches,
So that mens care is now to wear
 a Padlock on their Breeches.^35^

The author describes how young men are afraid to go to market or to the plough in case of being beset by the maidens. This, of course, reverses the usual pattern of sexual violence in which lone females might be assumed to be vulnerable to assault. The above stanza implies a sexually predatory dimension to the maidens’ activities; the padlock on men’s britches protects their ‘treasure’, but also brings to mind a chastity belt.

Ballads were not histories; the events described in these songs were not necessarily based on real life, and if they were rooted in fact, they inevitably shaped those facts to make a more engaging narrative. Nonetheless, one can see in these texts the repetition of some key anxieties. In each castration ballad, gender hierarchies are turned upside down, with women overseeing the sexual disempowerment and humiliation of sexually active men. The misrule which is described in each case participates in a tradition of charivari or festive disorder, in which pent-up frustrations are violently released. As Martin Ingram has demonstrated, charivaris blended quasi-judicial punishment with festivity and misrule. Crucially, such events both ridiculed and revelled in disruption of the established order:

Central to the symbolism of charivaris were notions of hierarchy, inversion, reversal, rule and misrule, order and disorder—the world turned upside-down. The most straightforward explanation of charivaris is that they stigmatized as ridiculous inversions of the ‘natural’ hierarchy. … Yet it is arguable that at a deeper level of psychology these customs reflected a sense of the precariousness or artificiality of that hierarchy; and that the laughter of charivaris bore witness to ambiguities and unresolvable conflicts in the ideal and actual social system.^36^

Just as the charivari offered release of social tensions through ‘explosive laughter’, the ballads above present castration as something funny, when it happened to other people. I will argue below that the ambiguities Ingram describes in the charivari were also visible in accounts of self-castration. In both, men’s emasculation is viewed as comically absurd. Nonetheless, it also reflects an underlying awareness of the precarity of patriarchal power, the constant need to re-construct masculinity in opposition to the existential threat posed by women and effeminate men.^37^ Viewed in tandem with stories of religious and judicial castration, these ballads demonstrate the disruptive potency of the gelding motif. Tales of castration emphasised the humiliation and ignominy associated with male impotency. Gelding was often a grotesque inversion of the natural order of things, in which women were subordinate to men, subjects to kings and Christians to scripture. Yet, this was not the only note sounded in accounts of castration. Stories of gelding often emphasised the dangerous nature of male desire. Castration, they intimated, became necessary when men were unable or unwilling to contain their sexual urges, and acted in socially, spiritually, or personally deleterious ways as a result. Furthermore, among the grotesquery emerged—sometimes at least—the possibility of making agency from emasculation, such that physical impotency might pave the way for spiritual potency. Against this confused and dynamic backdrop, accounts of self-castration emerged.

## Cuckolds and Jilts: Self-Castration as Psychodrama

When the alderman’s nephew made the fateful decision to castrate himself, he followed the dubious example of a Church father in rejecting his capacity for sexual sin. It is clear, however, that the life of this young man thereafter cannot have been an easy one. As the ballads above demonstrate, gelded men were often the butt of jokes. Moreover, they were excluded from the rituals of courtship and marriage which structured much of early modern life. What, then, can have induced this young man to expose himself to shame, ridicule and potential social exclusion? A clue to this question’s answer lies in Yonge’s assessment of his patient. While the young man suggests that religious fervour underlies his actions, Yonge is more convinced that ‘some disappointment in Love’ is to blame. In this sentence, Yonge pinpoints a psychodrama which seems to underlie many early modern accounts of self-gelding. That is, self-gelding is undertaken as an expression of emotional distress, and more specifically as a disavowal of the limited models of manhood available to young males in this period.

This theory is borne out by other accounts of self-gelding from the seventeenth and eighteenth centuries. Only a year before Yonge’s report, John Browne’s *Discourse of Wounds* related a similarly gruesome tale. This time, not one but two men were involved:

We had two in this our City of *Norwich* which endeavoured to castrate themselves upon the very thoughts of not marrying, mistrusting that if ever they should have any children, they could not maintain them: The first of which had taken out and cut off both his Testicles, but hereby occasioning such a flux of blood as was past his skill to stop, he sendeth for a Chirurgeon of our Town who speedily stops this and heals up the wound, and cures the Patient. The second not being so couragious, but entering upon his intended operation, could not with such dexterity act his part; but upon undertaking to take the first out, he occasions such a flux of blood as he thought would speedily have rewarded his bold attempt with death. Hence was forced to send for a Chirurgeon, who after having stopt the flux agglutinated the wound, and the Patient remains in very good health.^38^

Despite the danger which accompanied their activities, Browne did not present his patients as suicidal, nor as mentally ill. This attitude prevailed throughout medical and popular descriptions of men who gelded themselves. Although it was acknowledged that their reactions were extreme, they were explicable; every narrative of self-gelding contains a more or less detailed explanation of the factors which drove the protagonist to act in the way they had. This pattern of reporting probably owed something to the fact that suicide was a crime. Those who completed suicide, particularly in the first half of the seventeenth century, would have their property seized and their bodies buried outside church grounds.^39^ The sanctions placed on attempted suicides are less clear, but one would expect them to suffer social stigma and possibly exclusion from church worship. In addition, however, stories about self-gelding emphasise the perceived conflict between the protagonist’s ‘self’ and their intractable body. As in Yonge’s account, the young men in Browne’s story sought to sterilise themselves, but it was less clear here whether they also wished to avoid sex per se. As I discuss below, gelded men were widely believed to be not only infertile but incapable of sustaining an erection. Thus, one can read this episode as an act of extraordinary self-abnegation in which the pair seek to do away with both sex and sexual desire, freeing themselves from the anguish of experiencing lusts which they cannot responsibly satisfy. However, these protagonists’ specific emphasis on fathering children leaves open the possibility that they viewed gelding as a sort of contraceptive rather than a means of enforced celibacy. Adding to the complexity of the case, the right of gelded men to marry was in this period hotly contested. Since a primary purpose of marriage was procreation, one might assume that gelded men were excluded from this rite. However, Mary Frandsen and Helen Berry have both written at length about cases in which castrato singers successfully asserted their right to wed.^40^ The degree to which these men sought to remove themselves from the libidinal economy of courtship, sex and marriage is thus unclear.

Whatever their motivation, the extreme actions of the Norwich pair represented an attempt to assert their dominion over sexual desires which were incompatible with ‘higher’ economic concerns. In this respect, they too acted ‘Origen Secundus’, seeking to renounce the body in a way that would be deemed ‘couragious’ rather than insane. This narrative naturally required—or fostered—a dualist view of embodiment, in which the body and the mind ‘inside’ it were quite distinct, and capable of wanting different things. As the accounts above show, the conflict was easily framed as one between a moral ‘self’ and a body naturally inclined to vice, but this was not always the case. For instance, Charles Ancillon’s *Italian Love* (1704) tells of a young man who cut off his testicles and sent them to his mistress after finding that during an intimate encounter he was ‘so unhappy as not to be master of the instruments of his passion, which would not … obey him, but were all ice and snow, while his heart was on fire’.^41^ The specific complaint against the body in this case may be different, but the view of body and mind is the same. An unruly carapace fails to cooperate with the subject ‘inside’. This approach to embodiment is in stark contrast to a monist humoral view in which heats and emotions are experienced as intrinsic to both mind and body, with thoughts affecting physiology and vice versa. In depicting the body as potentially ‘disobedient’ to the mind, however, it also demonstrates how dualist approaches to embodiment still needed to accommodate the felt reality of bodily urges. This was not a simple case of the flesh versus the spirit, but of mind and body in constant—sometimes fraught—interaction. Moreover, I suggest that this view of embodiment was often improvisational in nature, such that these men’s desire to self-mutilate *created* a feeling of dissonance between mind and body as much as it stemmed from it. That is, self-castrators might be motivated in the first instance by emotional stressors. The desire to harm oneself was shaped by cultural influences such as the religious and judicial exempla discussed above, and these in turn fostered a notion of embodiment which allowed one to attack the body in defence of the ‘self’.

What were the emotional factors which might set this chain of events in motion? Yonge, Browne and Ancillon’s accounts all present different motivations based around the same theme of strife in or frustration with romantic relationships. All of these, however, were relatively unusual in focusing on thwarted male desire. The majority of self-gelders were motivated by a different fear—that of illimitable female desire. When men feared that they might be cuckolded, nagged or duped into bringing up illegitimate children, in some instances they turned to violence, not against their wives, but against themselves. As was often the case, this scenario appeared both in popular ballads and in medical texts, neither of which told the whole truth about any given incident. The ‘The Quaker’s Wife’s Lamentation’ (*c*.1684–1700), for instance, displays most of the hallmarks of self-gelding stories. A man who suspects his wife of infidelity removes his testicles, and in so doing, hopes to both shame his wife and assure himself of the paternity of any future offspring. His wife’s response to this curse is comical, but also underlines her libidinous nature:

Oh husband, husband, what have you done?
You’ve parted with Jewels were none of your’n,
But they were Jewels belonging to me,
For which I’d not take Gold nor Fee;
Them I delighted more to feel,
Than e’er I did my Spinning Wheel.
Ah! My dear Wife it was my fault
That I am Lame, and thou must Hault,
For had’st thou but prov’d true to me,
Then I had done the like to thee,
And if thou had’st been true my Girl,
I ne’er had parted with Natures Pearl.^42^

Printed in the late seventeenth century, this ballad seems designed in part to draw attention to the peculiarities of Quakers. At the end of the ballad, the husband suggests that his wife may get pregnant instead using a special powder which he has procured, which she may drink in a glass of wine. The ballad writer concludes that ‘I doubt the Cure will prove but Lame, / There’s nothing like to the Old Game’. The broader implication of this tale, however, is that self-mutilation may be an effective, if wrong-headed, response to female insubordination. Both the Quaker and his wife will be shamed when his gelding becomes common knowledge, and the community response to them will be akin to that levelled at cuckolds:

Ah my Dear Husband, you’re to blame,
To bring upon us so much shame,
For most men will both say, and swear
That you wid go to next Horn-Fair

The Quaker’s orchiectomy is thus both a reaction to his wife’s suspected infidelity and a means by which their marital dysfunction is publicised. Ironically, he has failed to prevent the outcomes he fears; his wife, under guise of the special powder, may still cheat and become pregnant, and he is still to be counted among the local cuckolds.

The *Lamentation* might appear comical, but its rhetoric was fairly subdued in comparison to similar tales one could find in medical and scientific texts. Among the earliest mentions of self-gelding was a case fleetingly related in Robert Burton’s *Anatomy of Melancholy* (1621), of a baker who ‘gelded himselfe to trye his wives honesty’.^43^ Over a century later, in 1741, Daniel Turner offered a more expansive version of a similar event:

A poor hypochondriac, or enthusiast rather, whether on a suspicion of his wife’s incontinency, and thereby to discover the truth, in case she brought him any more children, which was the common rumour, or to punish his own, I could never rightly learn: but so it fell out, that in a fit of melancholy, having shut himself up in his chamber, without any apparatus, he made shorter work, with one stroak of his razor, taking both testes and scrotum away together; Upon which so great effusion of blood from the arteries ran through the floor, as made the first discovery; the people below hastening up to him, perceived him in a manner expiring by the loss of blood, and calling presently for help …. he was rescued from the most imminent danger, and the wound, after digestion, being brought into a healing condition; the poor man ashamed of his late enterprise, nor daring to stir out of the house, removed his quarters by nights, and was never after heard of.

Benivenius tells of a Monk, who through a blind zeal to keep himself honest, cut off the penis close to the os pubis, from whence he was very near death by the great haemorrhage … de Abditis Akakia … of a poor baker, suspecting his wife’s incontinency, [who] cut off both his testes.^44^

The above accounts, like ‘The Quaker’s Wife’s Lamentation’, focused on paternity and the difficulty of knowing whether one’s children were one’s own. Moreover, each text described households in which the ideal patriarchal order had been disturbed, and men no longer had reliable control over their wives. This theme was likewise evident in a report originally by Montaigne, reworked in Ancillon’s eighteenth-century *Italian Love*, in which a woman’s nagging drove the protagonist to geld himself in a spectacular fashion:

[Montaigne] tells us of a certain *Peasant* in his neighbourhood, that made himself an eunuch for quite different reasons, which was for meer passion and anger against his wife; this good man, as soon as he came home, was received by his wife, who was jealous of him to an extravagance, and was continually tormenting him with the usual welcome, and said any thing against him that came uppermost, and as her jealousie furnished her with malicious abuses, he made no more ado, but immediately, with his scyth that he then had in his hand, whipt off those parts which gave her so much umbrage, and without any more ceremony threw them in the good woman’s face.^45^

There is little or no clinical or scientific utility to these stories. Instead, they prioritise a strong narrative. They emphasise the circumstances leading to the event and the personalities of the people involved, and they focus on lurid details such as the blood dripping through the floor. In all, they have a carnivalesque flourish not unlike the ballad tales of marauding maidens described above. Nonetheless, the stories they tell are psychologically complex, presenting self-castration as an issue of power within relationships rather than a matter of mental disorder. This sense of self gelding as a choice rather than a compulsion is what made it possible for medical writers to narrate such events in the language of popular literature, even while the treatment required afterwards justified both their inclusion in medical textbooks and the gruesomely frank language in which the whole process was described. In his *Anxious Masculinity*, Breitenberg comments on the baker’s castration related in the *Anatomy of Melancholy*:

Any psychological explanation—and clearly there is some psychological process at work here—must interpret the baker as having internalized the social system in which his identity is shaped and conferred. In his self-castration he enacts upon himself the vulnerability of men in patrilineal cultures since their identities depend to a large extent on the proper dissemination of property and status through women. The baker’s desire to maintain *control* over his own dissemination—even if it means castration—overwhelms even the dissemination itself.^46^

The self-castrators in these stories clearly wish to reassert control over the women with whom they are involved, primarily by enforcing their celibacy. If one’s husband is definitely infertile, then any pregnancy will be transparently the result of adultery, and women’s sexual activity is therefore proscribed. What makes these cases particularly interesting, however, is that the men involved choose to create this effect by gelding rather than simply by declining to have sex with their wives. One might conclude that the men involved believed that that they could still perform sexually without their testicles, but would not impregnate their partner—that is, they would be infertile but not impotent. However, this runs counter to popular beliefs about gelding and eunuchism in the early modern period. Occasionally, medical writers speculated that castrated men might be able to get an erection and possibly even have penetrative sex.^47^ In general, however, it was accepted that gelded men, like gelded animals, lost both the desire and the capacity to engage in heterosexual sex (though they were suspected of having homosexual encounters).^48^ Texts about eunuchs repeatedly asserted that eunuchs were created by Eastern or Turkish rulers in order to guard harems, because they could be trusted not to have sex with the women under their guardianship.^49^ Moreover, this view seems to be confirmed in the ‘Quaker’s Wife’s Lamentation’, in which the gelded Quaker is accused of leaving behind ‘a Thing that cannot stand’.^50^

If one accepts that the gelded man was widely believed to be impotent, then the decision of some men to geld themselves as a means of keeping their wives honest becomes a matter of symbolism as much as pragmatism. The above accounts make clear that in removing their generative potential, self-castrating men literalized their feelings of emasculation and powerlessness. In changing their bodies, they also sought to abdicate a masculine identity based on heteronormativity, patrilineage and patriarchal authority; a role whose requirements they found impossible to fulfil. In very broad terms, men were understood to be superior to women on religious grounds and on the basis of their temperament and anatomy, which were closely linked. As a result of this superiority, men had a greater right than women to hold property and status, and to pass both those things on down the patrilineal line. At the higher echelons, this system depended, as Breitenberg has demonstrated, on men’s ability to conduct relationships *through* as well as with women, securing homosocial bonds by the strategic use of marriage.^51^ Troublingly, however, this setup was contingent on the smooth working of patrilineage. The stories above, in which men are constantly anxious about their wives’ capacity to stray, demonstrate that patrilineage was in practice something of which it was very difficult to be assured. Of course, this situation also depended on the willingness of some men to have sex with women who were not their wives; in Turner’s account, it is suggested that both husband and wife might be unfaithful. In sum, men could never be entirely sure if the child to whom they passed on goods and status was actually their own. Moreover, as Ingram notes ‘women could never be dominated to the degree implied in the patriarchal ideal’, and expressed their non-compliance with this system in a vast range of ways, from nagging to outright cuckoldry.^52^ In the homosocial network which governed much of early modern economic, social and civic life, heterosexual reproduction was thus both the weak link and the most necessary component.

This weak link may have come under particular pressure during the early modern period. Tim Hitchcock and Michelle Cohen have identified ‘increasing anxiety about men’s sexual performance’ based in part on shifting medical understandings of gender, and more widely on demographic changes which saw men marrying later in life.^53^ Drawing from Lawrence Stone, Breitenberg similarly argues that the pace of social and economic change during the sixteenth and seventeenth centuries provoked increased attention to matters of gender identity and sexual ethics. The number of sexual offences brought to trial in the Elizabethan period increased dramatically, including defamation suits from women protecting their reputations.^54^ There was also a ‘steady stream’ of new bills passed by Parliament relating to fornication, bastardy and adultery.^55^ In the seventeenth century, some women published protestations about misogyny. These, along with tales of women cross-dressing, were the subject of angry reactions from men out of proportion to their actual incidence. As a whole, Breitenberg contends:

The period was decidedly nervous about social disorder in general and, at least from the perspective of men, that threat was all too easily located in misogynistic caricatures of women and in depictions of female sexuality as monstrous or destructive to themselves.^56^

In her *Vernacular Bodies*, Mary Fissel provides further evidence of what she characterizes as a ‘crisis in paternity’ in the second half of the seventeenth century (notably, the period from which all the above ballads are drawn). As was the case for accounts of castration, concerns about paternity appeared across a variety of textual genres. Ballads drew incessantly on the theme of men marrying a woman who gave birth a few months later, while medical texts pondered the question of maternal imagination and its influence on the unborn child.^57^ Overall, she argues, men were more worried than ever about the possibility that they were being cuckolded, or raising a child not their own.

In popular medical books, in ballads, and in political propaganda, paternity was in crisis. Medical books problematized any easy assignment of paternity, showing how easily disrupted paternal inheritance could be. In ballads, men were repeatedly deceived by women, cuckolded constantly and never sure about which children were truly theirs. On the national level, questions about monarchical succession quickly turned into scandalous rumours about the king’s mistresses and the paternity of his acknowledged illegitimate sons.^58^

Self-gelding narratives seem readily to fit into this climate. The men involved in each tale were keenly aware that masculine identity was premised on heterosexuality and paternity. Whether because the object of their affections declined to return them, or because their wives were felt to be untrustworthy, these aspects of masculinity could not be fulfilled. Self-castration offered a means to punish the women who disrupted this system. More than this, however, it provided a means by which men could repudiate the libidinal economy altogether; escaping the demands of masculinity by declaring themselves emasculate.

While their delivery differed widely, castration ballads and self-castration accounts shared an emphasis on the social, sexual and psychological motives behind castration and gelding. In attending to these motives, they show the importance of men’s generative capacity to the way in which they were perceived by others. Losing one’s testicles could be a horrifying mutilation, which might bring on humiliation and social exclusion. Equally, however, men who removed their own ‘stones’ were understood to do so as an act of agency rather than of madness. These men were not admired, but they were, to a certain extent, understood. When unable to adequately control their status in a compulsorily homosocial and heterosexual environment, they exerted the most extreme form of self-control.

## Conclusion

The history of self-gelding in the early modern period is a series of graphic, surprising and often blackly humorous anecdotes. As with many such histories, it is difficult to build a comprehensive picture from only a handful of incidents. To compound matters, those incidents are related in forms which clearly have a vested interest in emphasising certain, sensational aspects of what actually happened and omitting others. Nonetheless, the history of self-gelding is a worthwhile topic in its own right and as an aspect of the under-researched history of self-harm. In the modern medical literature, incidents of male genital self-mutilation are generally viewed as resulting from acute psychosis.^59^ A much smaller number of mutilations undertaken in clear consciousness are attributed to individuals forced to wait long periods for gender reassignment surgery.^60^ In early modern England, however, to geld oneself was not taken to signal either madness or a desire to become the opposite sex. Instead, these men were driven by the desire to exempt themselves from social systems in which they felt they had no viable place. There is, therefore, a question to be asked here about whether, and how, we might categorise such acts as self-harming; and if so, whether self-harm was understood as a pathology in the same way that it is today.

Men who engaged in self-gelding apparently cited the same reasons in many different forms. The masculine ideal was, they felt, one to which they could never attain. Their control over their own circumstances was insufficient to ensure the smooth running of the patriarchal and patrilineal structures on which ‘manhood’ was posited. Even more frustratingly, they found that their desire for sex persistently threatened their pursuit of an orderly lifestyle. Self-geldings occurred in part because men felt that they could not trust women, and in part because they did not trust themselves. That some men turned to self-gelding under these circumstances owed much to the status of castration in early modern culture. Historical, religious and judicial examples of castration influenced the way in which gelding was viewed as both shameful, degrading and potentially freeing. Finally, to castrate oneself also implied a feeling of dissonance between body and mind which resisted categorisation as simply monist or dualist. Men who saw fit to sever parts of their bodies clearly felt that there was a person ‘inside’ who was being betrayed by their bodily urges. Nonetheless, the fact of those bodily urges reiterated that the flesh could not be viewed in the same way as an automaton—if it could not be said to have an opinion, it might at least have an instinct. Acting ‘Origen Secundus’, or merely ‘disappointed in love’, men who turned the knife on themselves reveal much about the time in which they did so.

## Footnotes

1 James Yonge, *Currus Triumphalis, è Terebinthô. Or An Account of the Many Admirable Vertues of Oleum Terebinthinæ* (London: Printed for J. Martyn, 1679), 76–8.

2
*Ibid*., 76–8.

3
*Ibid.*, 78.

4 Gary Taylor, *Castration: An Abbreviated History of Western Manhood* (New York; London: Routledge, 2000), 56–7.

5 Daniel F. Caner, ‘The Practice and Prohibition of Self-Castration in Early Christianity’, *Vigiliae Christianae*, 1997, 51, 396–415, at 396.

6 Eusebius, *Eusebius: The Church History. A New Translation with Commentary*, trans. Paul L. Maier (Grand Rapids, MN: Kregel Academic, 2007), 213.

7
*Ibid.*

8 Mathew Kuefler, *The Manly Eunuch: Masculinity, Gender Ambiguity, and Christian Ideology in Late Antiquity* (Chicago: University of Chicago Press, 2001), 261.

9
*Ibid*.

10 John Anthony McGuckin, *The Westminster Handbook to Origen* (Louisville; London: Westminster John Knox Press, 2004), 6.

11 Thomas Stoughton, *The Christian’s Sacrifice* (London: William Jones, 1622), 190.

12 Ralph Venning, *The New Command Renew’d, or, Love One Another* (London: John Rothwell, 1652), 38.

13
*Ibid.*

14 The exception to the Catholic Church’s distaste for castration was the employment of *castrati* as Church singers; this phenomenon is discussed at length in Martha Feldman, *The Castrato: Reflections on Natures and Kinds* (Oakland: University of California Press, 2015).

15 Caner, ‘The Practice and Prohibition of Self-Castration in Early Christianity’, 402.

16 Ryan D. Giles, ‘The Miracle of Gerald the Pilgrim: Hagiographic Visions of Castration in the Liber Sancti Jacobi and Milagros de Nuestra Señora’, *Neophilologus*, 2010, 94, 439–50, at 441.

17 Jacqueline Murray, *Conflicted Identities and Multiple Masculinities: Men in the Medieval West* (London; New York: Routledge, 2013), 74.

18 Caner, ‘The Practice and Prohibition of Self-Castration in Early Christianity’, 403; Giles, ‘The Miracle of Gerald the Pilgrim’, 443.

19 Murray, *Conflicted Identities*, 75.

20 Giles, ‘The Miracle of Gerald the Pilgrim’, 442.

21 Murray, *Conflicted Identities*, 85.

22 See Feldman, *The Castrato: Reflections on Natures and Kinds*; Thomas A. King, ‘The Castrato’s Castration’, *Studies in English Literature 1500**–**1900*, 2006, 46, 563–83.

23 Martin Irvine, ‘Abelard and (Re)Writing the Male Body: Castration, Identity, and Remasculinization’, in Jeffrey Jerome Cohen and Bonnie Wheeler, eds, *Becoming Male in the Middle Ages* (New York: Garland Publishing, 1997), 87–106, at 89; Lila Yawn, ‘The Bright Side of the Knife: Dismemberment in Medieval Europe and the Modern Imagination’, in Anne Kirkham and Cordelia Warr, eds, *Wounds in the Middle Ages*, new edn, History of Medicine in Context (Farnham: Ashgate Publishing Ltd, 2014), 215–46.

24 Jacqueline Murray, ‘Sexual Mutilation and Castration Anxiety: A Medieval Perspective’, in Mathew Kuefler, ed., *The Boswell Thesis: Essays on Christianity, Social Tolerance, and Homosexuality* (Chicago: University of Chicago Press, 2006), 254–72, 256.

25 Irvine, ‘Abelard and (Re)Writing the Male Body’, 97.

26 Sean Eisen Murphy, ‘The Letter of the Law: Abelard, Moses, and the Problem with Being a Eunuch’, *Journal of Medieval History*, 2004, 30, 161–85, at 163.

27 Bonnie Wheeler, ‘Origenary Fantasies: Abelard’s Castration and Confession’, in Jeffrey Jerome Cohen and Bonnie Wheeler, eds, *Becoming Male in the Middle Ages* (New York: Garland Publishing, 1997), 107–28.

28 Irvine, ‘Abelard and (Re)Writing the Male Body’, 97.

29 Karin Selberg and Lena Wånggren, ‘The Dismemberment of Will: Early Modern Fear of Castration’, in Larissa Tracy, ed., *Castration and Culture in the Middle Ages* (Woodbridge: D.S. Brewer, 2013), 295–313, 299.

30
*Some Reasons Humbly Offer’d, Why Castration Instead of Death, May Prove to Be the Most Effectual Method of Punishing Persons Found Guilty of Robbery and Theft* (Dublin: 1731), 4–7.

31
*Ibid.*, 4–5.

32
*Ibid*., 5.

33 ‘Robin the Plow-Man’s Courage; Or, Harry the Millers Miserable Misfortune in Courting of Young Kate, Who Declared He Had Lost His Testicles, and Therefore Far Unfit for Wedlock’ (London: Printed for P. Brooksby, J. Deacon, J. Blare, and J. Back, 1675–96), <http://ebba.english.ucsb.edu/ballad/21321> (accessed on 28 September 2018).

34 ‘The Nine Maidens Fury / To The / Hartford-Shire Man. / Who Would Have Gelded Him, but That His Sweetheart Susan Coming in, Begged His Pardon’ (London: James Bissel, 1685–88), <http://ebba.english.ucsb.edu/ballad/21289> (accessed on 24 September 2018).

35 ‘The Hartford-Shire Mens / Fears of the Maidens Furies. / It Being an Answer to the Nine Maidens Attempt in Gelding the / Young-Man’ (London: James Bissel, 1685–88), <http://ebba.english.ucsb.edu/ballad/21290> (accessed on 24 September 2018).

36 Martin Ingram, ‘Ridings, Rough Music and the “Reform of Popular Culture” in Early Modern England’, *Past & Present*, 1984, 105, 79–113, at 96–97.

37 David Kuchta, *The Three-Piece Suit and Modern Masculinity: England, 1550**–**1850* (Berkeley: University of California Press, 2002), 10–12.

38 John Browne, *A Compleat Discourse of Wounds, Both in General and Particular* (London: E. Flesher for William Jacob, 1678), 327.

39 Michael MacDonald and Terence R. Murphy, *Sleepless Souls: Suicide in Early Modern England* (Oxford: Clarendon Press, 1993), 19–33.

40 Mary E. Frandsen, ‘“Eunuchi Conjugium”: The Marriage of a Castrato in Early Modern Germany’, *Early Music History*, 2005, 24, 53–124; Helen Berry, *The Castrato and His Wife* (Oxford: Oxford University Press, 2012).

41 Charles Ancillon, *Italian Love: Or, Eunuchism Displayed. Describing All the Different Kinds of Eunuchs; Shewing the Esteem They Have Met with in the World, and How They Came to Be Made so, Wherein Principally Is Examined, Whether They Are Capable of Marriage, and if They Ought to Be Suffered to Enter into That Holy State* *…* *Occasioned by a Young Lady’s Falling in Love with One, Who Sung in the Opera at the Hay-Market, and to Whom She Had like to Have Been Married*, 2nd edn (London: 1740 [1718]), 62.

42 ‘THE / Quaker’s Wives Lamentation / For the LOSS of / Her Husbands Jewels, / Who Gelded Himself (in Petticoat-Lane,) to Vex His Wife’ (London: James Bissel, 1684–1700), <http://ebba.english.ucsb.edu/ballad/21317> (accessed on 18 September 2018).

43 Robert Burton, *The Anatomy of Melancholy (1621)*, ed. William H. Gass (New York: New York Review of Books, 2001), 299.

44 Daniel Turner, *The Art of Surgery: In Which Is Laid down Such a General Idea of the Same, as Is* *…* *Confirmed by Practice*, 6th edn, 2 vols (London: C. Rivington, and J. Clarke, 1741), II, 279–80.

45 Ancillon, *Italian Love: Or, Eunuchism Displayed*, 63.

46 Mark Breitenberg, *Anxious Masculinity in Early Modern England* (Cambridge: Cambridge University Press, 1996), 16–17.

47 Nicholas Venette, *The Mysteries of Conjugal Love Reveal’d Written in French by Nicholas de Venette*, *…* *The 8th. Edition. Done into English by a Gentleman*, 2nd edn (London: 1707), 493–4.

48 Freitas speculates that the physical features of the *castrato*, whose early gelding affected multiple physical characteristics, may have rendered him homosexually attractive. However, there is little evidence of men castrated in adulthood having enhanced sexual value (Roger Freitas, ‘The Eroticism of Emasculation: Confronting the Baroque Body of the Castrato’, *The Journal of Musicology* 2003, 20, 196–249).

49
*The New Atlas, or, Travels and Voyages in Europe, Asia, Africa, and America* (London: J. Cleave and A. Roper, 1698), 20–1; Giovanni Benedetto Sinibaldi, *Rare Verities* (London: P. Briggs, 1658), 50.

50 ‘The Quaker’s Wives Lamentation’.

51 Breitenberg, *Anxious Masculinity*; see also Kuchta, *The Three-Piece Suit*, 51–70.

52 Ingram, ‘Ridings, Rough Music and the “Reform of Popular Culture” in Early Modern England’, 97.

53 Tim Hitchcock and Michelle Cohen ‘Introduction’ in Tim Hitchcock and Michelle Cohen, eds, *English Masculinities, 1660**–**1800* (London: New York: Routledge, 2014), 9.

54 Breitenberg, *Anxious Masculinity*, 19.

55
*Ibid*.

56
*Ibid.*, 21.

57 Mary Elizabeth Fissell, *Vernacular Bodies: The Politics of Reproduction in Early Modern England* (Oxford: Oxford University Press, 2004), 208–14.

58 Fissell, *Vernacular Bodies*, 197.

59 Helen Stunell et al., ‘Genital Self-Mutilation’, *International Journal of Urology*, 13, 1358–60; N. Eke, ‘Genital Self-Mutilation: There Is No Method in This Madness’, *BJU International*, 85, 295–8.

60 Stunell et al., ‘Genital Self-Mutilation’.

## Notes

Alanna Skuse is Wellcome Trust research fellow at the University of Reading, working on a project to trace the experiences of people whose bodies were altered by surgery in the early modern period. Her first book *Constructions of Cancer in early Modern England: Ravenous Natures*, is available via open access with Palgrave. She has also published on Shakespeare, disability, cosmetic surgery and Thomas Dekker.

